# The C‐terminal peptide plays a role in the formation of an intermediate form during the transition between xanthine dehydrogenase and xanthine oxidase

**DOI:** 10.1111/febs.13277

**Published:** 2015-04-13

**Authors:** Tomoko Nishino, Ken Okamoto, Yuko Kawaguchi, Tomohiro Matsumura, Bryan T. Eger, Emil F. Pai, Takeshi Nishino

**Affiliations:** ^1^ Department of Biochemistry and Molecular BiologyNippon Medical SchoolTokyoJapan; ^2^ Department of BiochemistryUniversity of TorontoONCanada; ^3^ Departments of Medical Biophysics and Molecular GeneticsUniversity of TorontoONCanada; ^4^ Campbell Family Institute for Cancer ResearchOntario Cancer Institute/University Health NetworkTorontoONCanada; ^5^ Department of Applied Biological ChemistryGraduate School of Agricultural and Life SciencesThe University of TokyoJapan

**Keywords:** endothelial cell damage, reactive oxygen species, xanthine dehydrogenase, xanthine oxidase, xanthine oxidoreductase

## Abstract

Mammalian xanthine oxidoreductase can exist in both dehydrogenase and oxidase forms. Conversion between the two is implicated in such diverse processes as lactation, anti‐bacterial activity, reperfusion injury and a growing number of diseases. We have constructed a variant of the rat liver enzyme that lacks the carboxy‐terminal amino acids 1316–1331; it appears to assume an intermediate form, exhibiting a mixture of dehydrogenase and oxidase activities. The purified variant protein retained ~ 50–70% of oxidase activity even after prolonged dithiothreitol treatment, supporting a previous prediction that the C‐terminal region plays a role in the dehydrogenase to oxidase conversion. In the crystal structure of the protein variant, most of the enzyme stays in an oxidase conformation. After 15 min of incubation with a high concentration of NADH, however, the corresponding X‐ray structures showed a dehydrogenase‐type conformation. On the other hand, disulfide formation between Cys535 and Cys992, which can clearly be seen in the electron density map of the crystal structure of the variant after removal of dithiothreitol, goes in parallel with the complete conversion to oxidase, resulting in structural changes identical to those observed upon proteolytic cleavage of the linker peptide. These results indicate that the dehydrogenase–oxidase transformation occurs rather readily and the insertion of the C‐terminal peptide into the active site cavity of its subunit stabilizes the dehydrogenase form. We propose that the intermediate form can be generated (e.g. in endothelial cells) upon interaction of the C‐terminal peptide portion of the enzyme with other proteins or the cell membrane.

**Database:**

Coordinate sets and structure factors for the four crystal structures reported in the present study have been deposited in the Protein Data Bank under the identification numbers 4YRW, 4YTZ, 4YSW, and 4YTY.

**Abbreviations**
ACPY‐NAD^+^: acetyl pyridine NAD^+^
AFR_25_: activity‐to‐flavin ratio, change in absorbance at 295 nm·min^−1^ divided by the absorbance at 450 nm at 25 °C
D/O ratio: dehydrogenase activity (absorbance change at 295 nm·min^−1^ in the presence of NAD^+^) divided by oxidase activity (absorbance change at 295 nm·min^−1^ in the absence of NAD^+^)
XDH: xanthine dxehydrogenase
XOR: xanthine oxidoreductase
XO: xanthine oxidase

## Introduction

Eukaryotic xanthine oxidoreductase (XOR) is a homodimeric protein with a relative molecular mass of 290 000 and is composed of independent subunits; each subunit contains one molybdopterin, two non‐identical [2Fe‐2S] clusters (designated as Fe/SI and Fe/SII; distinguished by redox potential and EPR signal) and one FAD as cofactors 1, 2. These centers are located in the C‐terminal 85‐kDa, N‐terminal 20‐kDa and intermediate 40‐kDa domains of the subunit, respectively 3, 4, 5. The enzyme catalyzes the oxidation of hypoxanthine to xanthine and xanthine to uric acid at the molybdenum center by oxidative hydroxylation and the reducing equivalents thus introduced are transferred rapidly via Fe/SI and Fe/SII to FAD, where the reduction of NAD^+^ or oxygen occurs 2, 6, 7.

Although XOR mediates purine catabolism, mammalian XOR exists in large quantities not only in the liver, but also in the mammary gland, kidney, intestine and other organs with vascular endothelial cells, such as the lung 8. Therefore, additional roles in those organs are likely. For example, mammalian XORs exist as two forms of the same gene product, xanthine dehydrogenase (XDH) and xanthine oxidase (XO), whereas other organisms have only the XDH form. XDH prefers NAD^+^ as an electron acceptor, whereas XO prefers molecular oxygen. In normal cells, XOR exists as its XDH form but can be converted to XO reversibly by the formation of disulfide bridges or irreversibly by limited proteolysis 9, 10, 11, 12, 13, 14. One example where the conversion is proposed to play a physiological role is in lactation 15, 16, 17, 18, 19. In addition, because the XO form produces reactive oxygen species, the conversion from XDH to XO is considered to be linked to anti‐bacterial defense 20, 21, 22 and various pathological events, such as ischaemia, inflammation and cardiovascular disease 23, 24, 25, 26, 27, 28. Thus, clarification of what triggers the conversion and how it proceeds is crucial for understanding the physiology of these pathological processes.

The enzymological and structural differences between bovine milk and rat liver XDH and XO are well defined 5, 14, 29, 30, 31. The conversion is considered to be physiologically significant, and not just an artifact of isolation, because the mechanism of conversion appears to be highly sophisticated 32. Briefly, in the crystal structures of the two forms, the conformation of the so‐called A‐Loop of Gln422 to Lys432 in the rat enzyme (Gln423Lys433 in the bovine enzyme) located near the FAD prosthetic group changes during the conversion of XDH to XO 5, 33, 34, thus dramatically altering the electrostatic environment and blocking access of the substrate NAD^+^ to its binding site. In rat XDH, the side chain of Asp428 (Asp429 in the bovine enzyme) is close to the flavin ring. Upon rearrangement of the A‐Loop, Asp428 moves away from the flavin ring and is replaced by the guanidinium group of Arg425. This rearrangement explains the differences in flavin redox potentials 31, 35, 36 and in the chemical environments revealed by artificial flavins 30, 37, 38, 39, 40. Another important difference between the two forms is the interaction of four specific amino acid residues, which form a unique amino acid cluster (consisting of Arg334, Trp335, Arg426, Phe549 in the rat enzyme and Arg335, Trp336, Arg427, Phe549 in the bovine enzyme) at the bottom of the FAD cavity; this cluster is tightly packed in the XDH form but disrupted in the XO form 33, 34.

The large number of cysteine residues incorporated into the amino acid sequence of XOR makes it very difficult to identify those that are involved in the conversion 32. The results of chemical modification of rat XOR with fluorodinitrobenzene led to the suggestion that Cys535 and Cys992 might be involved in the rapid phase of the biphasic XDH‐XO conversion 14. In the protein structure, Cys535 is located at the beginning of the long linker between the 40‐kDa and 85‐kDa domains, whereas Cys992 (Cys993 in human enzyme) lies on the surface of the 85‐kDa domain 41. On the other hand, linkage between the two domains was not observed in an electrophoretic analysis 16. Subsequent mutagenesis studies with rat XOR, however, showed that conversion of the C535A/C992R mutant in the presence of sulfhydryl residue modifiers is very slow, whereas the triple mutants C535A/C992R/C1316S and C535A/C992R/C1324S do not undergo conversion at all 41, suggesting that Cys535 and Cys992 are involved in the rapid phase and Cys1316 and Cys1324 in the slow phase of the modification reaction 14. It should be noted that the irreversible conversion of XDH to XO by trypsin involves limited proteolysis at the same linker peptide 5. It was proposed that triggering events, such as the formation of a disulfide bond between Cys535 and Cys992 or proteolysis of the linker, reorient Phe549 (also a part of the long linker), resulting in disruption of the four amino acid cluster mentioned above. Arg426 is then released from the cluster and moves the A‐loop that blocks the approach of NAD^+^ to the flavin ring of the FAD moiety, as well as changing the electrostatic environment, as described above. However, there was no definitive evidence for the formation of a disulfide bond between Cys535 and Cys992 by means of X‐ray crystallography. The role of Cys1316 and Cys1324 located in the C‐terminal portion is even less clear. In the crystal structures of the XDH forms of both bovine and rat XOR, the C‐terminal residues are seen inserted into the cavity of the FAD site; this is not the case in the corresponding XO forms 5, 41. Disorder in the preceding stretch of amino acids leading to a break in electron density made it impossible to decide whether the C‐terminal peptide in the binding site originated from the same subunit or from that of a neighboring molecule in the crystal lattice, although the latter with high probability would be a crystal artifact 41. Such flexibility and the slower modification rate of Cys1316 and Cys1324 by chemical modifying agents 14, 41 suggested that the C‐terminal peptide switches between inserted into and being outside the cavity. Cysteine modification is not likely to occur when the peptide is inserted but rather when it can freely move outside the binding site.

In the present study, we used site‐directed mutagenesis to construct a protein variant lacking the sixteen carboxy‐terminal residues (amino acids 1316–1331; CΔ‐protein variant) to further investigate the role of this C‐terminal peptide. We found that this variant appears to be an intermediate form; it functions as if a mixture of XDH and XO forms. When comparing the C‐terminal structures between the NADH‐bound forms of both the CΔ protein variant and the C535A/C992R/C1324S triple mutant, a remarkably stable XDH form, it became clear that insertion of the C‐terminal peptide into the dinucleotide‐binding cavity of its own subunit plays a crucial role in stabilizing the XDH form and generating a functional NAD^+^/NADH binding site.

## Results

### Expression and purification of the CΔ protein variant of recombinant rat liver XOR

**Figure 1:**
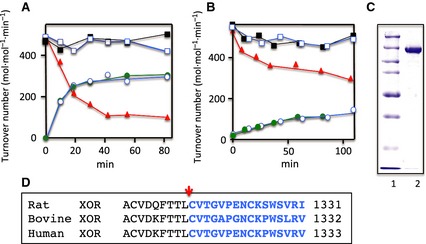
Time courses of change of activities of the purified CΔprotein variant XOR caused by DTT treatment. Wild‐type recombinant XOR (A: D/O = 1, AFR_25_ = 133, 1.0 × 10^−6^ m) and CΔ protein variant XOR (B: AFR_25_ = 109, D/O = 1, 4.2 × 10^−6^ m) were treated with 5 mm DTT in 50 mm KPB (pH 7.8) containing 0.4 mm EDTA at 25 °C. After incubation for various times, aliquots were withdrawn and the enzymatic activities were determined. The activities are shown as apparent turnover number (mol·min^−1^·mol FAD ^−1^) corrected for each enzyme's AFR_25_. The AFR_25_ of freshly prepared, fully active rat XOR enzyme is 200 29. Total urate formation activity (XDH plus XO activity) was determined by following the increase of absorbance at 295 nm in the standard assay mixture with xanthine and NAD ^+^ (closed squares) or ACPY‐ NAD ^+^ (open squares) as the substrate. XO activity was determined from the increase of absorbance at 295 nm without NAD ^+^ (red closed triangle). XDH activity was calculated from the increase of absorbance at 340 nm for NADH formation (green closed circles) or at 360 nm for ACPY‐ NADH formation (open circles). (C) SDS/PAGE: lane 1, marker proteins (Bio‐Rad) having molecular masses of 250, 150, 100, 75, 50, 37 and 25 kDa. Lane 2, purified protein variant XOR. (D) Comparison of C‐terminal amino acid sequences of rat, bovine, and human xanthine oxidases. Blue colored amino acids indicate the residues corresponding to the CΔ peptide of rat XOR.

Rat wild‐type XOR recombinantly produced in Sf9 cells retained most of its XDH activity and showed very low XO activity in the supernatant of the homogenate, as described previously 41, 42. XDH activity gradually decreased with a concomitant increase of XO activity during purification. For the CΔ protein variant, however, both XDH and XO activities were measured at almost equal levels in the supernatant solution of the homogenate. During chromatography, it then followed the pattern shown by the wild‐type enzyme, and XO activity increased with a concomitant decrease of XDH activity. At the end of purification, the enzyme exhibited only XO activity and the activity‐to‐flavin ratio (AFR_25_; i.e. change in absorbance at 295 nm·min^−1^ divided by the absorbance at 450 nm at 25 °C) values of the protein variant were in the range 100–160, indicating that 50–80% of the enzyme was in the active form (the AFR_25_ value of fully active enzyme is 200 29, 43 but can vary somewhat from preparation to preparation, probably as a result of the presence of desulfo‐enzyme) 42, 44, 45, 46, 47. The purified protein variant showed a single major band at ~ 150 kDa on SDS/PAGE (Fig. 1C), indicating that the increase of XO activity during purification was not a result of proteolysis but rather deletion of the C‐terminal peptide and disulfide‐bond formation, as confirmed below in the DTT treatment experiment. The spectra of the protein variant showed no significant difference from those of the wild‐type enzyme.

### DDT‐treatment of wild‐type and protein variant XOR

Purified wild‐type and CΔ protein variant rat XORs, both exhibiting mostly XO activity, were treated with 5 mm DTT at 25 °C for 1 h in 50 mm KPB (pH 7.8). The time course of the changes in the XO/XDH activity ratio of the wild‐type enzyme indicated that, although the sum of XO and XDH activity remained practically constant, the XO activity decreased, accompanied by an increase of XDH activity (Fig. 1A). This indicates that the wild‐type XOR can be re‐converted from XO to XDH form by DTT treatment, in agreement with previous studies 29. On the other hand, the conversion of the CΔ protein variant to its XDH form upon DTT treatment was limited; the purified enzyme showed mostly XO activity, although the D/O ratio [i.e. dehydrogenase activity (absorbance change at 295 nm·min^−1^ in the presence of NAD^+^) divided by oxidase activity (absorbance change at 295 nm·min^−1^ in the absence of NAD^+^)] of the DTT‐treated enzyme stayed well below 2 during prolonged incubation with DTT, indicating that the final mixture again contained similar amounts of XDH and XO forms (Fig. 1B). SDS‐gel analysis showed a single band; the protein variant enzyme was not cleaved by proteolysis at the linker between the two domains, a process responsible for irreversible conversion of XDH to XO 3. The CΔ protein variant was active toward both acetyl pyridine NAD^+^ (ACPY‐NAD^+^) and NAD^+^ (as in the case of wild‐type enzyme), suggesting that the redox potential of FAD was not significantly perturbed. Much longer incubation (days) was required for recovery of the XDH activity of the CΔ protein variant to the level of 50% of total activity in the presence of DTT and, during this time, the enzyme was partially converted to its desulfo‐form. We therefore used enzyme with a D/O ratio of ~ 1.5 for steady‐state kinetics studies. The data are consistent with the enzyme used being a mixture of XO and XDH forms, even after DTT treatment.

### Steady‐state kinetics of DTT‐treated and untreated CΔ protein variant

In agreement with a previous study on natural and DTT‐treated forms of rat liver XOR 29, Lineweaver–Burk plots for the recombinant forms of the wild‐type and the CΔ protein variant XOR showed parallel lines at a series of fixed concentrations of the second substrate (data not shown). The kinetic parameters are summarized in Table 1. The *K*_m_ values for xanthine were lower than 5 μm in all cases, being similar to the values obtained with the natural rat liver enzyme. The *V*_max_ values of the xanthine‐O_2_ activity for DTT‐untreated natural rat liver XO 29 and protein variant XO are also similar (i.e. 1030 and 960 mol·min^−1^·mol^−1^ of FAD, respectively. On the other hand, the *V*_max_ value for the xanthine‐O_2_ activity of DTT‐treated wild‐type XOR was 25–26% of that of untreated wild‐type XO; this could be explained by the fact that XDH itself still has oxidase activity, as in the case of chicken XDH, which is never converted to the XO form by proteolysis or sulfhydryl modifiers 2. Compared to these values, the *V*_max_ value for the xanthine‐O_2_ activity of the DTT‐treated protein variant is quite high, whereas that for the xanthine–NAD^+^ activity is very low. These results again indicate that the protein variant is a mixture of XDH and XO forms, even after DTT treatment.

**Table 1:** Steady‐state kinetic parameters of freshly prepared and DTT‐treated rat CΔ protein variant XOR. All experiments were carried out at 25 °C. The *V*_max_ value is corrected for the measured value of AFR, assuming that the AFR of fully active enzyme is 200 in the presence of NAD ^+^

Enzyme	*V*_max_ (X‐O_2_) mol·mol^−1^_·_min^−1^ (Δ295 nm)	*K*_m_ for O_2_ (μm)	*V*_max_ (X‐NAD^+^) mol^−1^·mol·min^−1^ (Δ340 nm)	*K*_m_ for NAD^+^ (μm)
DTT‐untreated	960 ± 31	49.7 ± 1.7	Not detected	–
DTT‐treated	625 ± 19	53.7 ± 4.4	222 ± 5	5.2 ± 0.3

### NADH reduction and guanidine‐HCl treatment of the CΔ protein variant

**Figure 2:**
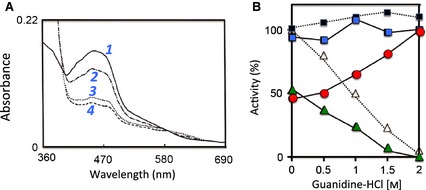
Time course of spectra of DTT‐treated C∆ protein variant immediately after mixing with NADH under anaerobic conditions and activity changes of the C∆ protein variant in the presence of low concentrations of guanidine‐HCl. (A) DTT‐treated C∆ protein variant protein was mixed with NADH as described in the Experimental procedures. The absorption spectra were recorded at various time intervals. Selected spectra are shown for clarity (*1*, solid line: initial spectrum; *2*, dash‐dotted line: immediately; *3*, dotted line: 10 min; *4*, dashed line: 89 min after mixing). (B) The enzyme (6.8 μm) was mixed on ice with various concentrations of guanidine‐HCl in 50 mm Tris‐HCl buffer (pH 7.8), 0.2 mm EDTA, 1 mm salicylate and 5 mm DTT and activities were measured within 5 min. CΔ protein variant: blue closed square, XDH plus XO activity determined by measuring urate formation in the presence of NAD ^+^ (∆295 nm); red closed circle, XO activity determined by measuring urate formation in the absence of NAD ^+^ (∆295 nm); green closed triangle, XDH activity determined by measuring NADH formation (∆340 nm); closed black square with dotted lines: bovine milk enzyme for comparison, XDH + XO activity; open triangle, XDH activity 48.

Because the CΔ protein variant shows only partial XDH activity even after prolonged DTT incubation, we performed a reduction experiment with excess concentration of NADH under anaerobic conditions. The reduction rate is slower than that of wild‐type XDH, as shown in Fig. 2A. The process is apparently biphasic, with a rapid phase (*k* = 8.32 min^−1^) followed by a much slower phase (*k* = 0.0792 min^−1^). The first phase accounts for ~ 50% of the final level of reduction. This is consistent with the idea that the enzyme exists as a mixture of two forms, with reduction of the XDH form being fast but that of the XO form being much slower.

We also investigated the effect of low concentrations of guanidine‐HCl on the relative amounts of the two forms. The experiment was carried out with DTT‐treated CΔ protein variant enzyme with a D/O ratio of 2 (i.e. ~ 50% of total urate formation could be assigned to XO). The enzyme was mixed with various concentrations of guanidine‐HCl in the presence of 5 mm DTT. As shown in Fig. 2B, the combined XDH and XO activity (total urate formation activity with NAD^+^) did not change, although the XO activity (urate formation in the absence of NAD^+^) was increased and the XDH activity (NADH formation activity) was lowered with increasing concentrations of guanidine‐HCl. The D/O ratio was stable for 90 min on ice, in accordance with previous results obtained with bovine milk XDH 48. The two forms are in equilibrium and guanidine‐HCl treatment presumably disrupts an amino acid cluster consisting of two arginine residues (Arg334 and Arg426), one tryptophan (Trp335) and one phenylalanine (Phe549) (residue numbers are those for rat XOR) by interfering with the π‐cation interaction that stabilizes it 33. After 2.0 m guanidine‐HCl treatment of the CΔ protein variant, up to 75% of the XO activity with D/O = 1.5 can be recovered by dialysis of the enzyme against a buffer containing 5 mm DTT but no guanidine‐HCl. This indicates that the conversion is reversible at low concentrations of guanidine‐HCl, as is the case with bovine milk XO 48. This suggests that, in solution, the two forms of XOR in the presence of < 2 m guanidine‐HCl are likely in a state of equilibrium. The enzyme can be inactivated irreversibly in the presence of guanidine at concentrations higher than 2 m; both XDH and XO activities are lost irreversibly, likely as a result of denaturation 48.

### Crystal structures of the CΔ protein variant enzyme

**Figure 3:**
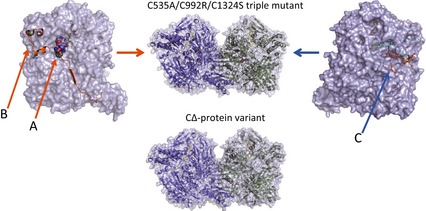
Crystal structures of a stable XDH form, triple mutant (C535A/C992R/C1324), and the CΔ protein variant of rat XOR. Space‐filling structures of the stable XDH form of the triple mutant are shown at the top and that of the CΔ protein variant at the bottom. Left: the monomer structure of the XDH form with FAD (in color‐code), the unique amino acid cluster (in space‐filling) and the long linker peptide (in brown). The model on the right is displayed rotated by 180° around the vertical two‐fold axis relating the two subunits; FAD (color‐coded), NADH (in blue) and the C‐terminal peptide (in brown) are also indicated. Arrows A (the amino acid cluster), B (the origin of the linker peptide) and C (the FAD cavity) in the C535A/C992R/C1324 mutant structure indicate the locations where it significantly differs from the CΔ protein variant structure (for further details, see Figs 4, 5, 6, 7).

The crystals of the CΔ protein variant of rat liver XOR were prepared in two ways: (a) crystallization of the protein directly after DTT treatment that in our experience is essential for crystallization to occur and (b) crystallization in the presence of DTT followed by extended soaks in mother liquor devoid of DTT to convert most of the protein to the XO form. Diffraction data to a resolution of 2.0 Å were collected from both samples (Table 2). The overall fold structures of bovine milk XDH and XO 5, as well as the triple mutant C535A/C992R/C1324S of rat liver XDH 41, which is completely resistant to conversion from the XDH to the XO form by sulfhydryl‐modifying reagents and therefore a model of stable XDH 41, are very similar. The same is true for the CΔ protein variant of rat liver XOR (Fig. 3); the only differences in conformation among all these proteins are found close to the FAD cofactor, in a unique amino acids cluster, and in the linker peptide between the FAD and Mo‐pterin‐ domains. All of the structures of the various rat liver proteins determined were of the demolybdo form 41, 42 because this was the only form for which amounts sufficient for crystallization experiments could be obtained. The absence of the molybdo‐pterin group, however, is not expected to have major conformational consequences at the FAD‐binding site 41, 42. In the present study, we focus only on the localized differences in conformation among all these proteins.

**Table 2:** Data collection and refinement statistics

	CΔ protein variant/crystallized with DTT	CΔ protein variant/crystal after removal of DTT	CΔ protein variant/NADH‐complex	C535A/C992R/C1324S triple mutant/NADH‐complex
Protein Data Bank code	4YRW	4YTZ	4YSW	4YTY
Data processing				
Space group	P2_1_2_1_2_1_	P2_1_2_1_2_1_	P2_1_2_1_2_1_	P2_1_2_1_2_1_
Unit cell axes (Å)	*a* = 98.5	*a* = 100.2	*a* = 98.5	*a* = 99.0
	*b* = 138.5	*b* = 139.1	*b* = 137.6	*b* = 137.4
	*c* = 222.2	*c* = 223.3	*c* = 222.1	*c* = 222.7
Wavelength (Å)	1.0	1.0	1.0	1.0
Resolution (Å)	50.0–2.06 (1.99–2.06)	29.0–2.30 (2.38–2.30)^a^	42.4–1.99 (2.06–1.99)	49.5–2.20 (2.28–2.20)
Unique reflections	206 606	134 745	195 400	153 088
*R*_sym_ (%)^b^	6.0 (20.2)	8.2 (36.8)	5.1 (18.8)	9.8 (32.0)
*I*/σ(*I*)	22.2 (5.4)	15.2 (3.2)	33.6 (5.2)	18.2 (7.1)
Completeness (%)	99.5 (96.4)	97.0 (98.5)	94.7 (90.1)	99.7 (96.0)
Redundancy	3.7 (3.6)	3.5 (3.3)	3.8 (3.4)	7.4 (7.3)
Refinement				
Resolution (Å)	43.3–2.0	29.0–2.3	42.4–1.99	49.5–2.20
Number of reflections	196 212	129 341	185 550	145 410
Number of test set reflections	10 396	5404	9850	7678
*R*_work_ c (%)	18.8 (22.1)	21.1 (26.6)	18.1 (20.3)	18.0 (18.7)
*R*_free_ (%)	22.9 (27.3)	26.4 (38.0)	21.9 (26.1)	22.1 (26.7)
Mean B‐factor (Å^2^)	30.0	35.1	24.0	21.2
Number of nonhydrogen atoms	20 716	20 055	21 751	21 735
Number of water molecules	638	105	1,639	1,433
RMS deviations				
Bond length (Å)	0.02	0.021	0.010	0.011
Bond angle (°)	2.07	1.89	1.21	1.31
Ramachandran plot				
Favoured (%)	96.1	95.7	97.4	97.1
Allowed (%)	2.9	3.6	2.2	2.4
Outliers (%)	1.1	0.7	0.4	0.5

a All values in parentheses refer to the highest resolution shell.

b *R*_sym_ = Σ_hkl_Σ_I_¦*I*_I_ – <*I*>¦/Σ_hkl_Σ_I_<*I*> where *I*_I_ is the *i*_th_ measurement and <*I*> is the weighted mean of all measurements of *I*.

c *R*_work_ = Σ_hkl_¦*F*_obs_ *– F*_calc_¦/*F*_obs_, where *F*_obs_ and *F*_calc_ are the observed and calculated structure factors, respectively, and the summation is over the reflections used for model refinement. *R*_free_ was the same as *R*_work_ for 5% of the data randomly omitted from the total data.

### Structure of the CΔ protein variant in the presence of DTT

**Figure 4:**
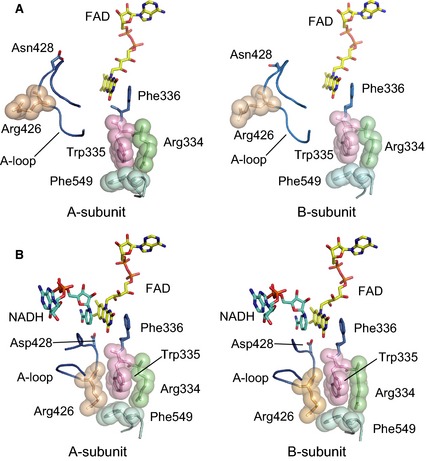
Active site structure around the FAD cofactor in the two subunits of the DTT‐treated CΔ protein variant in the absence and presence of NADH. (A) FAD, the A‐loop and the ‘unique’ amino acid cluster as seen in the absence of NADH. The ‘unique’ amino acid cluster consisting of Arg334, Trp335, Arg426 and Phe549 is shown in space‐filling mode, the A‐loop and Phe336 are in blue. (B), as in (A), although in the presence of NADH, which is shown as a blue stick model.

**Figure 5:**
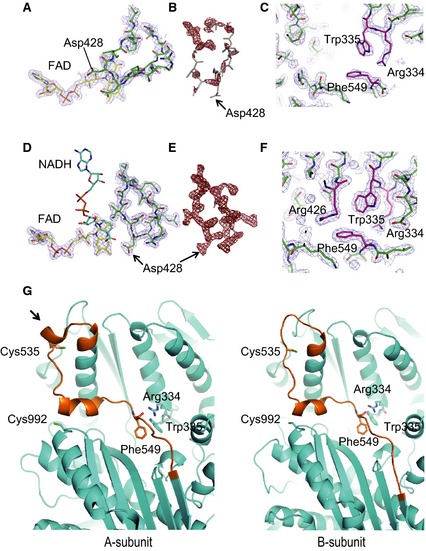
Comparison of electron density maps of CΔ variant in the absence of NADH (A–C) and in the presence of NADH (D–F). (A) 2*F*_o_ − *F*_c_ electron density contoured at 1.0 σ with the residues of the A‐loop and the FAD moiety modeled in. (B) 2*F*_o_ − *F*_c_ electron density contoured at 2.0 σ with the residues of the A‐loop displayed rotated. (C) 2*F*_o_ − *F*_c_ electron density contoured at 1.0 σ with the amino acid cluster residues modeled in. (D) 2*F*_o_ − *F*_c_ electron density contoured at 1.0 σ of the A‐loop and FAD moiety with residues modeled in. (E) 2*F*_o_ − *F*_c_ electron density contoured at 2.0 σ with the residues of the A‐loop rotated. (F) 2*F*_o_ − *F*_c_ electron density contoured at 1.0 σ with the amino acid cluster residues modeled in. Note that electron densities in (D) to (F) are much better defined than in the corresponding NADH‐free maps of (A) to (C), indicating reduced mobility. (G) Differences in conformation around Cys535, which is part of the long ‘linker loop’ connecting the FAD and molybdenum domains (brown color), and Cys992, as seen in the two subunits of the CΔ protein variant of NADH‐free rat XOR in the presence of DTT. In subunit A, the two cysteine residues are ~ 19 Å apart. The arrow points to a helical turn, which is not formed in subunit B.

In the CΔ protein variant, even after prolonged DTT treatment, the majority of the enzyme stays in the XO conformation, as described above. The corresponding crystal structure clearly shows the structural differences next to the FAD cofactor between this variant and the previously determined XDH‐form mutants 41; deletion of the C‐terminal peptide of 16 amino acids changes the structure of the ‘unique’ amino acid cluster and the position of the A‐loop (Fig. 4A). B‐factors of A‐loop residues are significantly higher and therefore the corresponding electron density weaker in the CΔ protein variant (Fig. 5A,B) than in any of the previously determined XOR structures, XDH as well as XO forms. This indicates increased flexibility of this loop with most Asp428 residues located far from the C6 position of the FAD isoalloxazine, as is typical for the XO form (Figs 4A and 5A). In addition, the two subunits displayed a partially disrupted ‘unique’ cluster, although not identical (i.e. Arg426 is no longer a part of the tight interactions that it forms with Arg334, Trp335 and Phe549 in native XDH) (Figs 4A and 5C). Arg426 follows the A‐loop, which has moved into the access path that NAD^+^ has to follow when approaching its FAD reaction partner. Thus, these results show that the CΔ protein variant assumes most of the conformational characteristics of the XO form. However, the two subunits are not completely identical, particularly near residue Cys535 in the long linker between the FAD and Mo‐domains (Fig. 5G). We discuss these features in more detail further below.

### Structure of the NADH complex

**Figure 6:**
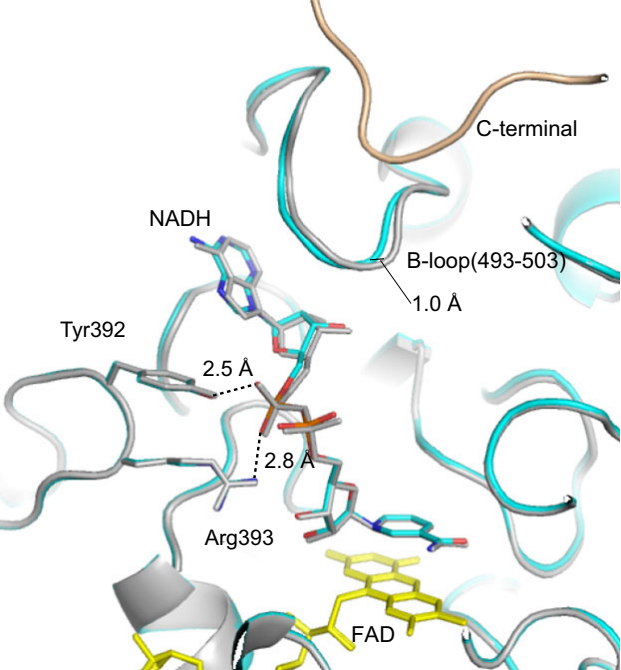
Crystal structures of the NADH complexes of the nonconvertible XDH‐form C535A/C992R/C1324S triple mutant and the CΔ protein variant of rat XOR. Models of the C535A/C992R/C1324S triple mutant (grey) and the CΔ protein variant (cyan) were superimposed based on their FAD cofactor (yellow). The respective NADH molecules (color‐coded), surrounding amino acids, and the C‐terminal peptide of the triple mutant (light brown), which is deleted in the CΔ protein variant, are also shown. Dashed lines indicate hydrogen‐bond interactions and the accompanying numbers give the inter‐atomic distances (Å).

To understand the role of the C‐terminal peptide in dinucleotide substrate binding, we determined the crystal structures of the NADH‐bound forms of both the CΔ protein variant (Figs 4B, 5D–F and 6) and, for comparison, that of the nonconvertible XDH‐form C535A/C992R/C1324S triple mutant (Fig. 6). In the crystal structure of the NADH‐bound triple mutant, the typical mode of NAD binding to flavoproteins was observed; the pyridine ring lies parallel to the isoalloxazine ring of FAD and engages in π‐π interaction (Fig. 6). The pyrophosphate group of NADH is contacted by Arg393 and Tyr392. This mode of binding by tyrosine and arginine residues is rather rare for this dinucleotide, although a related constellation has been observed in the case of the 2′‐phosphate group of NADP^+^ in its complex with isocitrate dehydrogenase 49. The corresponding Tyr392 residue in chicken XDH is covalently modified by 5′‐fluoro‐sulfonyl‐benzoyl adenosine (an affinity analogue of NAD^+^, resulting in loss of catalytic activity because of obstruction of NAD^+^ binding 50). The carboxyl group of Glu262 (not shown), whose orientation is shifted in the XO form of the CΔ protein variant, interacts with the 2′‐ and 3′‐hydroxyl groups of NAD^+^ ribose; the latter hydroxyl group also forms a hydrogen bond to Arg393. NADH interacts with the B‐loop (Leu493‐Met503) mostly via van der Waals contacts, supported indirectly by the C‐terminal peptide via stabilization of the B‐loop from behind (Fig. 6). Although the five amino acid residues from 1319 to 1324 were disordered in previous structures 41, and therefore no corresponding electron density could be observed, in the triple mutant NADH complex, the electron density for the whole C‐terminal peptide is well defined. It is clear that the C‐terminus is inserted into its own subunit rather than that of a neighboring molecule in the crystal lattice, thereby apparently regulating the binding of the electron acceptor NAD^+^.

In the crystal structure of the NADH complex of the CΔ protein variant, both subunits, assuming the XDH form, are almost identical, with the exception of the chain around Cys535. This part of the protein was indistinguishable in conformation from that of the NADH‐free form shown in Fig. 5G (data not shown for the NADH‐bound form).

Figures 4B and 5D–F illustrate two subunits and only one subunit, respectively, showing the position of the A‐loop, the position of Asp428 and the well‐packed amino acid cluster. Note that the electron density of the A‐loop (Fig. 5D,E) is higher than that found in the NADH‐free form of the CΔ protein variant (Fig. 5A,B) and the position of Asp428 is now close to C6 of the isoalloxazine ring (Figs 4B and 5D). Because the enzyme, based on its catalytic activity, was clearly a mixture of XDH and XO forms in solution, at the high concentration of NADH (20 mm) used in the preparation of the complex, both subunits of the enzyme appear to slowly shift to their XDH form in the crystal. This also is consistent with the results of reduction experiments shown in Fig. 2, when the protein variant could be fully reduced by prolonged incubation with a 50 : 1 surplus of NADH. Superposition with the structure of the NADH‐bound form of the C535A/C992R/C1324S mutant showed no major difference other than a maximum shift of ~ 1 Å at the B‐loop (Fig. 6), probably reflecting the lower affinity of the CΔ protein variant for NADH.

### Changes in the crystal structure of the CΔ protein variant after removal of DTT

**Figure 7:**
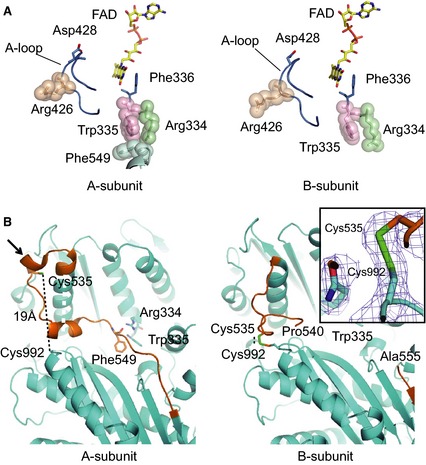
Structural features around the FAD cofactor and Cys535 of the CΔ protein variant after removal of DTT. (A) Arrangement of the cofactor FAD, the A‐loop and the ‘unique’ amino acid cluster in the two subunits of the CΔ protein variant of rat XOR in an oxidizing environment. The ‘unique’ amino acid cluster consists of Arg334, Trp335, Arg426 and Phe549 and is shown in space‐filling mode; the A‐loop and Phe336 are in blue. (B) Structural features around Cys535, which is part of the long ‘linker loop’ connecting the FAD and molybdenum domains (brown color), and Cys992, which lies on the surface of the molybdenum domain, in the two subunits of the CΔ protein variant of rat XOR in an oxidizing environment. In subunit A (left, the two cysteine residues are ~ 19 Å apart. A black arrow points to a helical turn, which is lost after conversion to XO. In subunit B (right), disulfide formation brings the two cysteine residues close together. Inset: 2*F*_o_ − *F*_c_ electron density map contoured at 1.0 σ covering this disulfide bond.

When crystals of the CΔ protein variant are incubated for an extended period of time in mother liquor devoid of DTT, formation of the disulfide bond between Cys535 and Cys992 described in the Experimental procedures is expected, leading to a shift of the enzyme's conformation towards the XO form. Indeed, this is what is seen in the CΔ‐mutant structure (Fig. 7), although, in subunit A, slight residual electron density can still be observed at the position of the XDH form. The electron density map clearly shows the structural differences between the two subunits; deletion of the C‐terminal peptide of 16 amino acids influences the structure of the ‘unique’ amino acid cluster and the position of the A‐loop (Fig. 7A). The electron densities of A‐loop and amino acid cluster in this subunit, however, were difficult to distinguish unequivocally as a result of their disordered structure, a feature typical of the XO form 5, 34.

In addition, the vast majority of subunits A displayed a partially disrupted cluster (i.e. Arg426 is no longer a part of the cluster that it forms in native XDH with Arg334, Trp335 and Phe549). It now follows the A‐loop, which has moved into the access path for NAD^+^‐binding, interfering with the approach of the pyridine ring of NAD^+^ to its FAD reaction partner. In subunits B (Fig. 7A), the cluster is almost completely disrupted; not only Arg426, but also Phe549 has moved away and is not visible in the density map as a result of a disordered linker peptide. The A‐Loop has left the FAD site and blocks the NAD^+^ binding pathway.

Although disulfide‐bond formation between Cys535 and Cys992 in the XO form has been proposed previously 14, 41, our observation in subunit B is the first experimental evidence of a disulfide bond linking two cysteine residues (Fig. 7B, and electron density in inset). In this structure, the one helical turn (indicated by an arrow in Fig. 7B, subunit A) at the beginning of the long linker connecting the 40‐kDa and 85‐kDa domains is lost and the Cys535 residue, also at the beginning of the linker, moves toward Cys992, which, in the XDH form, is 19 Å away, as discussed previously 32 and also shown in Fig. 5G. It is interesting to note the marked contrast between subunits A and B (Fig. 7B). The former adopts a conformation very close to the native XDH structure (i.e. without loss of the turn of the mobile α‐helix) and the two cysteine residues (fully 19 Å apart) are obviously not forming a disulfide bond, although some distortion of the unique cluster is visible in this structure.

## Discussion

Deletion of the C‐terminal peptide of rat XOR converted some of the enzyme from the XDH to the XO form. The crystal structures of the protein variant indicated that the two subunits of the homodimer can become asymmetric under certain conditions, reflecting the solution measurements showing the enzyme to be a mixture of XDH and XO. The asymmetry must lead to changes on the surface of the protein molecules that allow repetitive selective packing in the crystal lattice [one dimer (in the same orientation)/asymmetric unit]. Otherwise, the differences would be washed out through superposition. All other crystal structures of XOR enzymes determined so far are of pure XDH or XO forms and therefore do not show such differences between subunits. The new protein variant structures are in good agreement with its catalytic properties. Even prolonged DTT treatment does not lead to identical subunits, as described above. Non‐identical behavior of two subunits has also been found previously at the molybdenum center of XOR, based on kinetics 51 and X‐ray studies 52.

As shown by a previous study of native bovine milk XOR 48 and confirmed by the present experiments with the CΔ protein variant, the XDH to XO conversion can already occur in relatively low concentrations of guanidine‐HCl (< 2 m), where the overall protein structure remains intact. The extent of the conversion does not depend on time (the state is stable for 1 h on ice) but does depend on the concentration of guanidine‐HCl, whose removal caused return to the XDH form, suggesting that there is a reversible equilibrium between the two forms. On the other hand, disulfide formation between Cys535 and Cys992, which can be clearly seen in the electron density map of the present crystal structure of the CΔ protein variant without DTT, goes in parallel with the complete conversion to XO, resulting in the disruption of the amino acid cluster and an associated movement of the A‐loop, which are changes identical to those observed upon proteolytic cleavage of the linker peptide.

With deletion of the C‐terminal peptide shifting the enzyme to the XO form, it has to be assumed that the peptide not only plays a role in NAD^+^ binding, but also stabilizes the XDH conformation by inserting itself into the dinucleotide‐binding cavity. The XO conformation can be shifted to the XDH form by exposing the enzyme to NADH, although long incubation times are required. This mirrors the results of the spectrophotometric reduction experiment shown in Fig. 2A. Based on the results of the C‐terminal peptide deletion together with the experiments with guanidine‐HCl, we consider the transition between the XDH and XO forms to be in thermodynamic equilibrium, with a stable intermediate possible. The existence of such an intermediate had been postulated based on spectroscopic measurements 53.

Our results indicate that the the XDH to XO shift is influenced by whether the C‐terminal peptide is inserted into the FAD cavity or not. In addition to cysteine residues, which can undergo disulfide bond formation, the C‐terminal peptide contains also hydrophobic and positively‐charged amino acids. These residues could interact with other proteins or membranes, thereby shifting the XDH to XO equilibrium in favor of the latter by engaging the peptide outside the FAD cavity.

**Figure 8:**
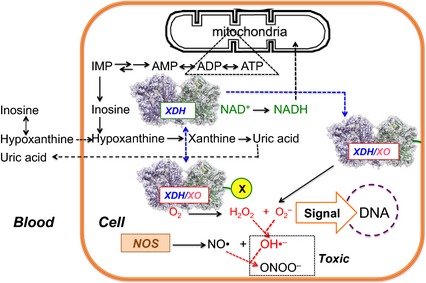
Schematic illustration of possible roles of the intermediate type of XOR, in which the C‐terminal peptide interacts with other proteins (X) or the cell membrane. The substrate hypoxanthine or its precursor inosine, which in erythrocytes are in equilibrium catalyzed by purine nucleoside phosphorylase, are transported to capillary endothelial cells where XOR exists in the intermediate form generated by the interaction of the C‐terminal peptide with other proteins (X) or the cell membrane. XDH‐type XOR produces NADH that can be used for oxidative phosphorylation in mitochondria; however, XO‐type XOR produces ROS (H_2_O_2_ + O_2_^−^), which could act as signaling mediators for DNA synthesis or generate toxic species such as OH• or ONOO ^‐^. Reaction of O_2_^−^ with NO• also decreases NO•.

XOR not only exists in large quantities in the mammary gland, but also is present in various other organs, including endothelial cells of vascular capillaries 8. Clinical studies of XOR inhibitors in hyperuricemic patients have indicated that a sufficiently high concentration of a drug in the bloodstream is more important in humans than in rodents (i.e. a low concentration of a potent XOR inhibitor was still effective in decreasing uric acid and allantoin in rodents but not in humans) 54. This suggests that, in humans, in contrast to rodents, substantial amounts of XOR are present in organs other than the liver 54. Physiological or pathological roles for the observed XDH to XO transition have been proposed for lactation in mammals 15, 16, 17, 18, 19 or in processes such as post‐ischemic reperfusion injury 23, 24, 25, 26 and the induction of endothelial cell damage 28, 55, 56, 57. Possible pathological roles of partial conversion of XDH to XO are illustrated in Fig. 8.

Although XOR is mostly found in the cytosol of liver cells 58, its interaction with the membrane of capillary endothelial cells has also been reported 59, 60, 61, 62. It is tempting to speculate that the C‐terminal peptide may be involved in such interactions. Also in vascular endothelial cells in other organs, where XOR may again be associated with membranes or other proteins with affinity for the C‐terminal peptide (indicated with an ‘X’ in Fig. 8), conversion to the intermediate type could be triggered or at least aided by these interactions. We do not want to suggest that interactions between the C‐terminal peptide and cell components are the only way the XDH to XO transition can be influenced and intermediate forms of XOR generated. The long linker peptide or the ‘unique’ cluster are other obvious points of attack for XOR regulation. Especially under energy consuming conditions, hypoxanthine is provided to endothelial cells in large amounts via the bloodstream from other organs such as muscle or brain, which do not contain XOR 63, 64, enabling the generation of large amounts of reactive oxygen species (H_2_O_2_ + O_2_^−^) in these cells. The results reported in the present study suggest that even partial conversion of XDH to XO, which does not require proteolysis, may be a significant source of such damaging molecules.

## Experimental procedures

### Materials

Baculovirus AcNPV DNA, transfer vector BacVector^™^ 2000 (Novagen, Madison, WI, USA), *Spodoptera frugiperda (Sf9)* cells, culture media and fetal calf serum were obtained and used as described previously 41, 42. The resin used for enzyme purification by folate affinity chromatography has also been described previously 43, 65, 66. The polyclonal antibody used in these experiments was raised in our laboratory against purified rat liver XOR 43. All other chemicals were of reagent grade.

### DNA manipulations and site‐directed mutagenesis

A baculovirus‐insect cell system was employed for expression of wild‐type 42 and mutant XOR 41. Site‐directed mutagenesis was performed according to well‐established methods 41, with minor modifications. The oligonucleotide was hybridized to single‐stranded pUC119NX7 and introduced into *Escherichia coli* strain HB 101 (Takara Shuzo, Tokyo, Japan). The resultant double‐stranded vector was isolated and digested with *Not*I and *Xba*I. The fragment obtained was ligated with pRXD203 that had been digested with the same restriction enzymes, and the DNA fragment encoding the mutant enzyme was excised by *Nhe*I and ligated into the baculovirus transfer vector pJVP10Z. The direction of the cDNA was identified by DNA sequencing. The carboxy‐terminal deletion protein variant (CΔ protein variant), missing the last 16 amino acid residues, was obtained by changing the Cys1316 codon to a Stop codon. The forward mutagenesis primer for the PCR product was 5′‐AGGGGATCATAAAGATCTCCGTACGG‐3′ (a new *Bgl*II restriction site is underlined), and the reverse primer was 5′‐GGTGGTACCGCTAGCTACAGGGTGGTGAACTGGTC‐3′ (the *Kpn*I site, *Nhe*I site and Stop codon are underlined). Because XDH is a rather large molecule, the vector site of the XDH plasmid was prepared using the two‐step replacement method. For the first step, the small PCR product was restriction‐digested at the *Bgl*II and *Kpn*I sites. This PCR product of 491 bp from the *Bgl*II to the *Kpn*I site was inserted into the pRX203 vector restriction‐digested at the *Bam*HI site at 705 bp and the *Kpn*I site at the 3′‐multicloning site. For the second step, the product of the first step was digested with *Not*I at the site located at the 5′‐noncoding‐multicloning site derived from the T_3_ promoter and at the *Eco*RV site of the vector. This vector contained the Stop codon instead of Cys1316. The insert was prepared to restrict the plasmid pRXD203, which contained full‐length wild‐type XDH 42 at the *Not*I and *Eco*RV sites (3.5‐kbp insert). The CΔ protein variant expression plasmid was prepared by ligation at the *Not*I and *Eco*RV sites using the vector containing a Stop codon instead of the Cys1316 codon and the 3.5‐kbp insert at the same sites described above.

### Expression and purification of XOR and its mutants using the baculovirus‐insect cell system

XOR and its mutants were expressed in a baculovirus‐insect cell system as described previously 42. The purification protocol was also as described previously 42. Recombinant active XORs and demolybdo‐dimeric XORs were separated by affinity column chromatography. The enzymes were incubated with 5 mm DTT for 1 h at 25 °C to generate the XDH form of XOR, followed by gel filtration to remove excess DTT, if necessary.

### SDS/PAGE

SDS/PAGE was performed as described by Laemmli 67 using 10% polyacrylamide gels. Protein markers, consisting of a mixture of recombinant proteins (10–250 kDa), were purchased from Bio‐Rad (Tokyo, Japan).

### Enzyme assays

All standard enzyme assays were carried out as described previously 41. Total urate formation activity (XDH plus XO activity) was determined by following the increase of absorbance at 295 nm in the standard assay mixture with xanthine and NAD^+^ or ACPY‐NAD^+^ as substrates. XO activity was measured in terms of the increase in absorbance at 295 nm without NAD^+^. XDH activity was calculated from the increase in absorbance at 340 nm (NADH formation). In addition, we also used ACPY‐NAD^+^ as an electron acceptor; the reaction was then followed at 360 nm using an extinction coefficient of 9.1 mm ^−1^·cm^−1^ for the reduced form 68. The concentration of native or protein variant recombinant active rat XOR was determined by spectrophotometry using an extinction coefficient of 35.8 mm ^−1^·cm^−1^, whereas a value of 33 mm ^−1^·cm^−1^ was used to measure the concentration of the demolybdo‐form of the enzyme 42. The dehydrogenase‐to‐oxidase ratio (the D/O ratio) as defined by Waud and Rajagopalan 69 was determined as reported 12. The AFR value was also measured as described previously 42.

### Steady‐state kinetics of xanthine‐NAD and xanthine‐O_2_ activity

Most reactions were performed at 25 °C in 50 mm potassium phosphate buffer (pH 7.8), 0.4 mm EDTA under air‐saturated conditions; the exception was the determination of xanthine‐O_2_ activity, for which variable concentrations of oxygen were used in an Aminco–Chance‐type cuvette equipped with an enzyme injector. All of the enzymes used for kinetic studies had been purified over a folate affinity column 43; the concentrations (final) and AFR_25_ values of the enzymes used were in the ranges 1.76–6.6 nm and 90–146, respectively. Xanthine concentration varied between 0.82 and 12 μm, ensuring that the substrate concentration remained sufficiently high to allow proper measurements, even in the presence of significant amounts of inactive forms of the enzyme (varying between 27% and 55%). Five different concentrations of electron acceptors were measured; they were in the ranges 6.8–450 μm for NAD^+^ and 69–1290 μm for oxygen. The *V*_max_ value was corrected for the measured value of AFR_25_, assuming that the AFR_25_ of the fully active XO form of the enzyme is 200 39, 43. The resulting double‐reciprocal plots of reaction velocity and xanthine concentration showed good linearity.

### Anaerobic reduction of the CΔ protein variant with NADH

The experiment was performed under anaerobic conditions as described previously 42. The CΔ protein variant enzyme (5 μm and D/O ratio ~ 2) in 50 mm KPB at pH 7.8 was anaerobically mixed with NADH at 25 °C. The final concentrations of added NADH were 50 times higher than the corresponding enzyme concentrations.

### Crystallization of the CΔ protein variant of XOR and collection of diffraction data

The enzyme used for crystallization was further purified on a gel filtration column of TSKSW3000XL (Tosoh Co., Tokyo, Japan) just before crystallization to remove aggregated enzyme possibly still present after folate affinity chromatography. As described previously 42, the recombinant XOR was expressed in multiple forms and the enzyme used for crystallization was the demolybdo‐dimeric form. The enzyme was concentrated to 8 mg·mL^−1^ in buffer A [mixture of 15% v/v of 50 mm pyrophosphate buffer (pH 8.5) and 85% v/v of 50 mm potassium phosphate buffer pH 7.4] 43 and incubated with 5 mm DTT for 60 min at 25 °C. Crystals of the protein variant enzyme were grown by vapor diffusion, equilibrating a mixture of 1 μL of protein and 1 μL of reservoir solution containing 9–11% poly(ethylene glycol) 8000, 0.8–1.2 m lithium formate (CryoPro/4M; Hampton Research, Aliso Viejo, CA, USA), 5 mm DTT, 1 mm sodium salicylate, 0.4 mm EDTA, 15% glycerol and 40 mm Hepes (pH 6.2) against 1.5 mL of reservoir solution. Crystals of the NADH complex were prepared by soaking crystals with mother liquor containing 20 mm NADH followed by incubation at room temperature for 15 min. Crystals of the enzyme were flash‐frozen with their mother liquor as a cryo‐protectant and mounted in cryo‐loops. Several crystals of the CΔ protein variant, after crystallization with DTT, were moved to fresh reservoir solutions free of DTT for at least 1 week at 20 °C to convert them to the XO‐form. Diffraction data were collected at 100 K on beamline NW12A of KEK (Tsukuba, Japan) using radiation of 1.0 Å wavelength and a Quantum 210 area detector (Area Detector Systems Corporation, Poway, CA, USA) and reduced with the help of hkl2000 70. The crystal structures were determined by molecular replacement techniques using molrep 71. The atomic models were built using coot 72 and refined using ccp4, version 6.1 73. The same software was used for superimposing molecules. Cystal structures were generated with pymol 74.

## Author contributions

ToN designed the experiments, conducted the experiments, and wrote the paper. KO performed the experiments and data analyses of crystal structures, and generated images. TM and YU designed and performed the experiments. BTE performed diffraction data analyses. EFP performed diffraction data analyses, wrote the MS and contributed funding. TaN designed the experiments, analyzed data, wrote the MS and contributed funding.
